# Electrocardiogram markers predicting ischemic stroke after acute coronary syndrome

**DOI:** 10.1016/j.ijcrp.2025.200500

**Published:** 2025-08-22

**Authors:** Matilda Hurskainen, Juho Tynkkynen, Leo-Pekka Lyytikäinen, Terho Lehtimäki, Kjell Nikus, Jussi Hernesniemi

**Affiliations:** a Faculty of Medicine and Health Technology, Tampere University, Tampere, Finland; b Centre of Vascular Surgery and Interventional Radiology, Tampere University Hospital, Tampere, Finland; c Tays Heart Hospital, Tampere University Hospital, Tampere, Finland; d Department of Clinical Chemistry, Fimlab Laboratories, Tampere, Finland; e Finnish Cardiovascular Research Center Tampere, Tampere, Finland

**Keywords:** Acute coronary syndrome, Myocardial infarction, Ischemic stroke, ECG, Risk factors

## Abstract

**Background:**

Patients with coronary artery disease (CAD) have increased risk of ischemic stroke (IS). Our aim was to screen for significant electrocardiogram (ECG) features for IS risk in patients treated for acute coronary syndrome (ACS).

**Methods:**

This retrospective registry study is based on 7760 ACS patients treated in Tays Heart Hospital (2007–2018) with follow-up for incident IS until December 31st^,^ 2020. ECGs recorded during ACSs were analysed by the Marquette™ 12SL™ ECG Analysis Program version 24. Preliminary screening for ECG features was conducted using age- and sex adjusted Cox regression analysis and corrected by multiple testing (Bonferroni method). Highly correlated variables were excluded from the final age-, sex- and atrial fibrillation (AF)/atrial flutter (AFL) adjusted Cox regression and subdistribution hazard (SDH) multivariable analyses.

**Results:**

From 7760 patients, 489 (6.3 %) suffered IS during a median follow-up of 5.7 years (IQR 3.1–8.8). In the final multivariable model, the main risk factors were premature ventricular complexes (PVCs) or aberrantly conducted complexes in AF/AFL (SDH, 2.01 [1.22–3.31]), left ventricular (LV) hypertrophy (LVH) by Sokolow-Lyon criteria (SDH, 1.52 [1.12–2.06]), S wave amplitude in lead V4 (SDH, 1.13 [1.05–1.21]) and negative P wave peak time in lead V2 (SDH, 1.12 [1.02–1.23]). T wave amplitude in lead V6 (SDH, 0.78 [0.69–0.88]) and T wave duration in lead aVL (SDH, 0.85 [0.78–0.92]) showed an inverse association with IS risk. The continuous variables correspond to 1 SD.

**Conclusions:**

ECG markers demonstrating LV dysfunction, LVH and atriopathy associate with IS risk after ACS, although external validation is still required.

## Highlights

• This study brings forth updated applicable ECG variables related to ischemic stroke risk after acute coronary syndrome.
• Dysfunction of the left ventricle presenting in ECG alterations is linked to ischemic stroke risk.
• S wave amplitude in lead V4 is associated with ischemic stroke risk.
• Atriopathy in ECG increases ischemic stroke risk independent of atrial fibrillation.

## Graphical abstract

**Figure:**
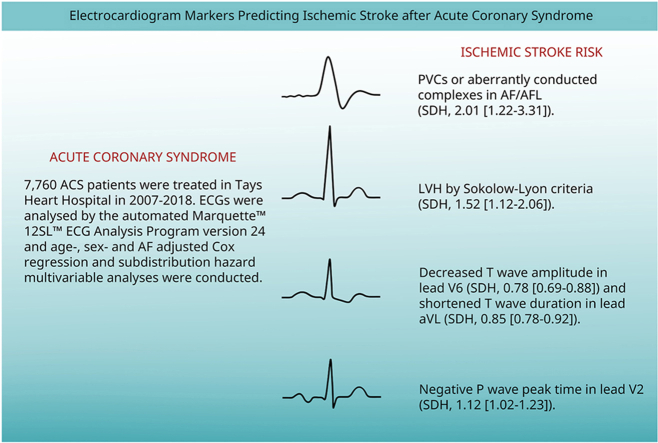

## 1 Introduction

Ischemic stroke (IS) is a known complication after acute coronary syndrome (ACS) and atrial fibrillation (AF) and atrial flutter (AFL) are well established risk factors for IS [1,2]. Atrial cardiopathy, also known as atriopathy, presenting with left atrial enlargement (LAE), has also been shown to relate to increased IS risk even in the absence of AF [[3], [4], [5]]. Different alterations in the P wave reflecting atriopathy, such as abnormal P wave terminal force in lead V1 (PTFV1), increased maximum P wave area (PWA) and P wave duration (PWD), abnormal P wave axis (aPWA) and prolonged P wave peak time (PWPT) in lead V2 have also been shown to associate with increased IS risk [3,[5], [6], [7], [8], [9]]. In addition, complete P wave disappearance (CPWD), advanced interatrial block (AIAB) and atrial premature beats (APBs) are linked to the risk of future AF and, further, IS [[10], [11], [12], [13]].

ACS has been proven to increase IS risk especially during the first years, but also long term, even for over 10 years [[14], [15], [16]]. The role of myocardial infarct (MI) location on IS risk, however, is a more disputed issue. Some studies have linked anterior MI to an increased risk of left ventricular (LV) dysfunction and cardioembolic (CE) stroke, yet others found no difference in infarct location and IS risk [[17], [18], [19], [20], [21], [22]].

Heart failure (HF) and LV hypertrophy (LVH) have previously been associated with IS risk [[23], [24], [25]]. ECG is rarely normal in HF and AF, Q waves, LVH and a widened QRS complex are possible alterations reflecting it [26]. A downsloping ST segment with asymmetric T wave inversion in the lateral leads (I, aVL, V5, V6) known as the “strain pattern” is thought to be an early marker of LV dysfunction and has been linked to increased IS risk [27]. Additionally, premature ventricular complexes (PVCs) have been associated with increased risk of IS [28].

No extensive analysis of a wide range of ECG markers associating with IS risk following ACS has previously been conducted. The aim of this retrospective registry study was to screen for different automatically detected ECG markers predicting IS after ACS.

## 2 Methods

This study is based on the MADDEC (Mass Data in DEtection and prevention of serious adverse events in Cardiac patient) retrospective registry study recording mass data of all cardiac patients treated in Tays Heart Hospital, which is the sole provider of specialized and tertiary cardiac health care in the region of Pirkanmaa, Finland [29]. Data for the registry is collected from multiple sources including electronic health records (EHR), written patient records and the prospectively updated KARDIO registry, which records structured patient data. The KARDIO registry data is maintained by treating nurses and physicians using an online tool. As a result, the MADDEC database comprises high-quality phenotype information of all patients treated for ACS [29]. The study design was approved by the Pirkanmaa Hospital district and due to the nature of the study, informed consent was not required. It also complies with the Declaration of Helsinki on the ethical principles for medical research. This study design and baseline phenotype data collection have been thoroughly described previously [16,29,30].

All patients undergoing coronary angiography for ACS between January 1st^,^ 2007 and December 31st^,^ 2018 and residing in Pirkanmaa were included. For the purpose of this study, only the first ACS for each patient was selected as the index event even if multiple events were recorded. Patients with an ECG recorded maximum of seven days prior to one day after angiography (95 % of ECG recorded the same or the following day) for ACS were included in the study. Only the most recent ECG in the timeframe per patient was used in the analyses, even though multiple ECGs were recorded. Thus, the study population consisted of 7760 patients.

### 2.1 Follow-up and end-point definitions

Patients were followed from the index ACS event (including hospitalization period) until the patient suffered an IS (due to any cause), or died, until December 31st of 2020. Only the first IS for each patient was analysed. Follow-up information on incident IS was collected by an in-depth review of EHR containing hospital discharge diagnoses, all written medical records from specialized health care and written death certificates detailing the cause of death and the circumstances leading up to the event. This thorough review was conducted for every patient to discover all true IS events. Patients who suffered only a transient ischemic attack (TIA) or other condition, such as Moyamoya disease, were excluded from the analyses, as well as IS occurring only prior to ACS. IS events were subtyped by the TOAST (Trial of Org 10172 in Acute Stroke Treatment) criteria and additional phenotypic data from the time of the IS event was also collected similarly by reviewing the medical records.

### 2.2 Automated ECG analysis

**Fig. 1:**
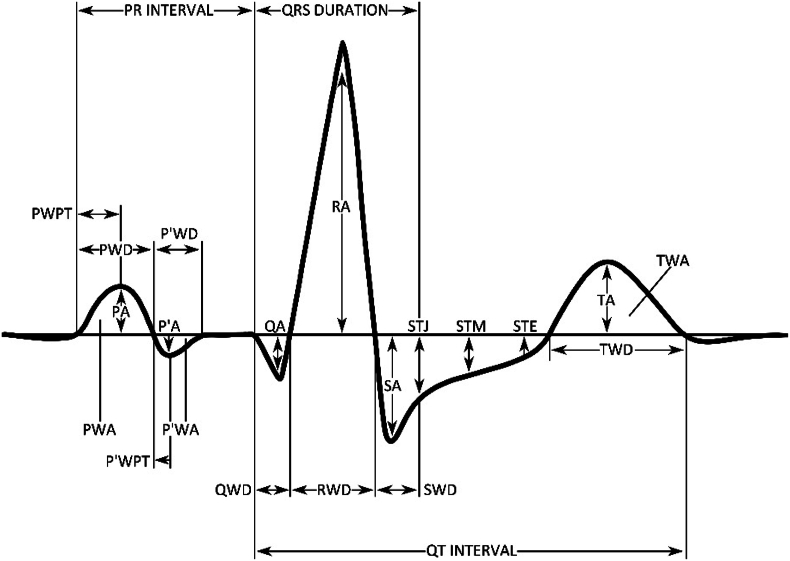
ECG variables presented visually. The picture is created by Matilda Hurskainen. P-value categories: P wave: P wave amplitude (PA), negative P wave amplitude (P’A), P wave peak time (PWPT), negative P wave peak time (P’WPT), P wave duration (PWD), negative P wave duration (P’WD), P wave area (PWA), negative P wave area (P’WA), PR interval. QRS complex: QRS duration, Q wave amplitude (QA), Q wave duration (QWD), R wave amplitude (RA), R wave duration (RWD), S wave amplitude (SA), S wave duration (SWD), QRS balance (maximum R amplitude – maximum S amplitude), QRS deflection (maximum R amplitude + maximum S amplitude), QT interval. ST level: ST elevation in J point (STJ), ST elevation in middle point (J point + 1/16 of the average PR interval) (STM), ST elevation in endpoint (J point + 1/8 of the average PR interval) (STE), minimum ST amplitude (minimum of STJ or STM). T wave: T wave amplitude (TA), T wave duration (TWD), T wave area (TWA), special T amplitude (minimum of either T amplitude or T amplitude-STE).

All ECGs screened were digitally stored in the MUSE® Cardiology Information System and processed using the Marquette™ 12SL™ ECG Analysis Program version 24 (GE Healthcare, Wauwatosa, WI), which forms a median ECG presentation of the most prevalent complexes in the 10s recording captured in the standard 12-channel ECG after denoising/preliminary filtering of artefacts. The ECG paper speed was 50 mm/s with 10 mm/mV size. From this median ECG, different parameters were calculated representing different ECG features such as P wave or QRS morphology (Fig. 1). Differing complexes presented in the standard 10s ECG recording were excluded from the median ECG interpretation and interpreted separately.

### 2.3 Definitions of ECG variables

Fig. 1 illustrates the electrical activity of a single heartbeat with relevant ECG variables. The P and T wave configuration may wary and be either monophasic (positive or negative) or biphasic (positive and negative). Thereby, P′ and T′ denote the negative components of said waves. Negative P wave peak time (P’WPT) refers to the duration from the start of the negative P wave to the peak, whereas P wave area (PWA) is the area above the baseline. P wave full area was calculated by adding PWA to the negative P wave area (P’WA) below the baseline. By definition, Q and S wave are negative deflections, and their amplitudes measured from the baseline are represented as positive values. ST level (both depression and elevation) was measured at three different points: J point, middle point (J point + 1/16 of the average PR interval) and endpoint (J point + 1/8 of the average PR interval). T wave amplitude (TA) was measured from the baseline and T wave duration (TWD) refers to the duration from the start of the T wave to the end of the T wave. LVH according to the Sokolow-Lyon Criteria was calculated as S wave in V1 + the R wave in V5 or V6 > 35 mm. PVCs or aberrantly conducted complexes refer to one or more premature ectopic shaped heart beats.

### 2.4 Statistical analysis

Automated analysis by the ECGs by the Marquette™ 12SL™ ECG Analysis Program version 24 was conducted, after which all categorical variables with low prevalence (<0.5 %) and non-biological or auxiliar summary statement related variables, such as a statement “abnormal ECG” or left/right parenthesis, were excluded. This resulted in 725 applicable ECG variables.

Next, a screening for features significantly associating with IS risk after ACS was performed to all the 725 variables using age- and sex adjusted Cox regression analysis. Variables depicting P wave morphology were screened separately after excluding ECGs presenting with AF/AFL (n = 660). The screening results were corrected by the Bonferroni method assuming 500 independent tested hypotheses. A threshold of p ≤ 0.05/500 = 0.0001 was used for variables to be considered for multivariable models.

Overlapping features were filtered out in order to control collinearities. For this, all variables were grouped based on clinical view into site/phenomenon specific groups: variables depicting the morphology of P waves, QRS complex, ST level, S wave, T waves or AF/AFL and LVH. Next, variables within these groups were individually tested for their correlation by Pearson correlation coefficient (r) and variables with high (>0.5 or < −0.5) correlation compared to the most representative variable of each group (lowest p-value in screening phase) were excluded. Also, every group's most representative variable was tested for correlation separately.

The final age-, sex- and AF/AFL adjusted multivariable analyses were conducted by Cox regression model with Schoenfeld residuals test for proportional hazard assumption (p < 0.05 indicating violation) and verified by subdistribution hazard (SDH) model. SDH was used to account for the competing risk of death possibly precluding the observation of IS, since this patient population has increased mortality. The homoscedasticity of the variables was verified by examining the partial residuals. Since the means were close to zero, the variance of errors was deemed constant. Therefore, Robust standard errors were not used. These analyses were done separately for ECGs without AF/AFL (n = 7100) also excluding the variables relating to AF/AFL and including P wave variables. Sex-stratified analyses were not performed due to insufficient statistical power in the smaller subgroups. The multivariable models were additionally adjusted for the CHA_2_DS_2_-VASc Score as a continuous variable, without the age related variable due to significant correlation.

Results for continuous variables (except age and heart rate) are presented after Z standardization (mean values centered to zero), and, thus, all the subsequent hazard ratios (HRs) denote the risk associated with 1 SD increase. This was done to enable direct comparison of different ECG variables with varying units of measurement. A p-value of <0.05 was considered statistically significant. The analyses were performed by SPSS software version 28 (IBM, Armonk, NY) and by R software version 4.1.3 (packages haven, survival, cmprisk).

## 3 Results

### 3.1 Demographics of the study population

The mean age of the study population (n = 7760) was 68.7 years (±11.8 SD) during hospitalization for ACS and 65.8 % (n = 5109) of the patients were men. The median follow-up time was 5.7 years (IQR 3.1–8.8). The majority (81.1 %, n = 6296) of ACSs were MIs and two thirds (65.2 %, n = 5059) were treated with percutaneous coronary intervention (PCI) and 10.5 % (n = 816) with coronary artery bypass grafting (CABG). In total, 489 of the 7760 ACS patients suffered an IS during the follow-up time. ACS subtype was not significantly associated with the risk of IS. Baseline characteristics of the study population are provided in Table 1. Baseline characteristics were missing for <7 % for LV ejection fraction (LVEF), <5 % for cancer and smoking status and <1 % for the rest of the variables. The small proportion of missing variables is due to the MADDEC registry being actively maintained by medical professionals.

**Table 1:** Baseline characteristics at the index event (recorded during hospitalization for acute coronary syndrome).

	All patients (N = 7760)
Age, years (SD)	68.7 ± 11.8
Sex, male % (N)	65.8 (5109)
Diabetes (any type) % (N)	26.0 (2017)
Hypertension % (N)	61.5 (4772)
Dyslipidemia % (N)	58.0 (4500)
Chronic Kidney Disease % (N)	6.9 (537)
Valvular Heart Disease % (N)	7.3 (566)
Atrial Fibrillation or Flutter % (N)^a^	19.4 (1.503)
Peripheral Artery Disease % (N)	8.3 (645)
Cancer % (N)	9.1 (707)
Dementia % (N)	2.7 (211)
Smoking % (N)^b^	44.8 (3479)
Chronic Obstructive Pulmonary Disease % (N)	7.0 (542)
Previous Stroke or Transitient Ischemic Attack % (N)	8.8 (684)
Previous Myocardial Infarction % (N)	17.3 (1345)
Previous PCI % (N)	10.3 (801)
Previous CABG % (N)	8.1 (626)
Previous ICD % (N)	0.3 (25)
Left Ventricular Ejection Fraction % (SD)	51.5 ± 11.8
Status during admission	
Haemoglobin (g/l) (SD)	130.2 ± 15.9
Creatinine (μmol/l) (SD	88.2 ± 58.2
Killip Classification for Heart Failure % (N)	
I	77.7 (6028)
II	14.1 (1098)
III	6.1 (476)
IV	1.9 (149)
Acute Coronary Syndrome Subtypes % (N)	
Unstable Angina Pectoris	18.9 (1467)
Non-ST-Elevation myocardial Infarction	48.5 (3761)
ST-Elevation Myocardial Infarction	32.6 (2532)
Treatment Modality % (N)	
PCI	65.2 (5059)
CABG	10.5 (816)
Conservative	24.3 (1885)

Data missing in <7 % for LVEF, <5 % for cancer and smoking and <1 % for all other variables.

Percentages are valid percentages. Continuous variables are mean and ± is standard deviation. Categorical values are frequencies.

Abbreviations: PCI, percutaneous coronary intervention; CABG, coronary artery bypass grafting; ICD, implantable cardioverter-defibrillator.

a History of atrial fibrillation or flutter or atrial fibrillation or flutter observed during hospitalization.

b Current or history of smoking.

### 3.2 ECG markers associated with the risk of IS in multivariable analyses

There was no data missing in ECG variables, since every ACS patient had an ECG taken during admission that was analysed by the automated Marquette™ 12SL™ ECG Analysis Program. According to the multivariable model, categorical ECG variables associating with increased risk of IS were PVCs or aberrantly conducted complexes in AF/AFL (SDH, 2.01 [95 % Cl, 1.22–3.31], p = 0.006) and LVH by Sokolow-Lyon criteria (SDH, 1.52 [95 % Cl, 1.12–2.06], p = 0.007) in addition to the AF/AFL variables (Table 2). PVCs analysed in the multivariable analysis instead of PVCs or aberrantly conducted complexes in AF/AFL showed no statistical significance in IS risk (p > 0.05). The prevalence of PVCs or aberrantly conducted complexes was higher (11,2 % n = 162/1443) in patients with current or previous AF/AFL compared to the prevalence of PVCs without (6,3 % n = 372/5878) AF/AFL.

**Table 2:** ECG factors in multivariable analysis associating with ischemic stroke after acute coronary syndrome (N = 7760).

Variable	HR (95 % CI)	p-value	SDH (95 % CI)	p-value	% (N)	Mean ± SD
Ten-year increase in patient age	1.46 (1.33–1.61)	<0.001	1.24 (1.14–1.35)	<0.001		68.69 ± 11.77
Sex (male)	1.04 (0.86–1.26)	0.700	1.12 (0.92–1.37)	0.240	65.8 (5109)	
Ten beats/minute increase in heart rate	1.08 (1.02–1.15)	0.007	1.03 (0.97–1.10)	0.320		70.23 ± 15.18
Previous or current AF/AFL	1.41 (1.10–1.81	0.007	1.38 (1.12–1.71)	0.003	19.4 (1503)	
AF in the ECG^a^	1.39 (1.03–1.87)	0.031	1.37 (1.01–1.86)	0.045	7.5 (585)	
LVH by Sokolow‐Lyon criteria	1.52 (1.13–2.06)	0.006	1.52 (1.12–2.06)	0.007	6.0 (462)	
PVCs or aberrantly conducted complexes in AF/AFL	2.00 (1.21–3.30)	0.007	2.01 (1.22–3.31)	0.006	1.3 (103)	
PVCs^b^	1.13 (0.78–1.64)	0.505	1.08 (0.74–1.56)	0.700	5.6 (432)	
S wave amplitude in lead V4 (mV)^d^	1.14 (1.05–1.23)	<0.001	1.13 (1.05–1.21)	0.001		0.72 ± 0.49
ST level elevation in endpoint in lead aVR (mm)^c^,^d^	1.10 (0.98–1.23)	0.111	1.02 (0.93–1.13)	0.650		−0.05 ± 0.54
T wave amplitude in lead V6 (mV)^d^	0.81 (0.71–0.91)	<0.001	0.78 (0.69–0.88)	<0.001		0.02 ± 0.20
T wave duration in lead aVL (ms)^d^	0.84 (0.77–0.91)	<0.001	0.85 (0.78–0.92)	<0.001		175.70 ± 67.67
Multivariable analysis without present AF/AFL in the ECG (7,100).						
P wave full area in lead V1 (μV∗ms)^d^	0.91 (0.83–1.00)	0.043	0.92 (0.84–1.01)	0.074		−0.95 ± 8.59
Negative P wave peak time in lead V2 (ms)^d^	1.14 (1.04–1.25)	0.005	1.12 (1.02–1.23)	0.020		19.89 ± 33.08

a Included into the multivariable model without the overlapping variable “Previous or current atrial fibrillation or flutter”.Previously or during the hospitalization for ACS diagnosed atrial fibrillation or flutter.

b PVCs in ECG without AF/AFL were additionally analysed in the multivariable model replacing the variable “PVCs or aberrantly conducted complexes in AF/AFL”.

c ST elevation was measured in three points: J point, middle point (J point + 1/16 of the average PR interval) and endpoint (J point + 1/8 of the average PR interval).

d HR and SDH values corresponding to one standard deviation increase in exposure variable.Abbervations: HR, hazard ratio; SDH, subdistribution hazard; SD, standard deviation; ECG, electrocardiogram; AF, atrial fibrillation; AFL, atrial flutter; LVH, left ventricular hypertrophy; PVC, premature ventricular complex; AFL, atrial flutter.

Continuous variables shown to increase IS risk after ACS were S wave amplitude (SA) in lead V4 (SDH, 1.13 [95 % Cl, 1.05–1.21], p = 0.001) whereas TA in lead V6 (SDH, 0.78 [95 % Cl, 0.69–0.88], p < 0.001) and TWD in lead aVL (SDH, 0.85 [95 % Cl, 0.78–0.92], p < 0.001) were associated with lower risk of IS (Table 2). These previous ECG variables correspond to 1 SD difference. Altogether, the results were similar comparing Cox regression and SDH analyses as can be seen in Table 2.

P’WPT in lead V2 (SDH, 1.12 [95 % CI, 1.02–1.23], p = 0.020) also slightly associated with IS risk after ACS in the analyses conducted without ECGs presenting with AF/AFL (n = 7100), (Table 2). These ECG variables correspond to 1 SD difference. Otherwise, the results stayed similar in both the Cox regression and SDH analyses. For Cox regression analyses, no violations of the proportional hazard assumption were found in Schoenfeld residuals test (p < 0.05 indicating violation).

Adjusting the multivariable model with the CHA_2_DS_2_-VASc Score as a continuous variable, without the age related variable (significant correlation), resulted in unchanged results as presented in the Supplement Table 1.

## 4 Discussion

This contemporary retrospective registry study establishes several different ECG features from a large number of variables (725) identified by the automated Marquette™ 12SL™ ECG Analysis Program version 24 related to IS risk after ACS. Two main pathologies depicted in ECG arise: markers demonstrating LVH/LV dysfunction (PVC's or aberrantly conducted complexes in AF/AFL, LVH and lateral alterations in T wave) and atripathy (P’WPT in lead V2), both of which may lead to the formation of CE [3,5,7,8,25,[31], [32], [33], [34], [35], [36]]. The results stayed unchanged after adjusting for the CHA_2_DS_2_-VASc Score as a continuous variable. Considering the widespread use of ECG in clinical practise, our study brings forth updated ECG parameters for future use in identifying high risk patients. To the best of our knowledge, there is no similar research with such vast range of ECG variables investigating IS risk.

We found PVCs or aberrantly conducted complexes in ECG presenting with AF/AFL and adjusted for AF/AFL to more than double the risk of IS after ACS. Presently, the pathophysiological mechanism behind PVCs and IS risk is not fully understood. PVCs have been linked to LV dysfunction, HF, and further, CE formation, which could explain the increase in IS risk. [23,36]. On the other hand, PVCs were not intrinsically associated with IS risk in our study suggesting the role of PVCs or aberrantly conducted complexes to be more significant in AF/AFL. For comparison, in a study by Agarwal et al., PVCs in routine ECG increased the risk of IS by almost 38 % without AF/AFL [28]. The role of PVCs and aberrantly conducted complexes in IS risk needs to be further studied to fully understand the impact, taken into consideration how common especially PVCs are as an incidental finding in the 12-lead ECG. Based on our study, it cannot be concluded whether the IS risk is due to PVCs or to aberrantly conducted, broad complex beats. These are different electrophysiological and clinical entities, but they are not always easy to differentiate in the ECG.

The prevalence of PVCs or aberrantly conducted complexes was also higher amongst patients with previous or current AF/AF, corresponding to previous research [37,38]. In a study by Kim et al., PVCs were associated with increased risk of both future AF and IS [38]. In theory, retrograde ventriculo-atrial conduction occurring in PVCs replicates ectopic atrial beats and is considered to lead to the development of AF later on [38].

HF as well as LVH are known risk factors for IS independent of ACS [[23], [24], [25]]. LVH is associated with cardiac disease, including cardiomyopathy. It also increases the risk of AF, which can be asymptomatic, paroxysmal or go undetected [39]. HF, LV dysfunction and AF are also possible complications after MI and have previously been linked to the risk of CE stroke [40,41]. In AF/10.13039/100010164AFL, thrombosis forms in the left atrium, whereas in LV dysfunction and HF it may form in the LV leading to 10.13039/100021407CE. Supporting this, we established LVH by Sokolow-Lyon criteria to increase IS risk by up to 50 %. A study by Antikainen et al. showed LVH by Cornell voltage or product, although not by Sokolow-Lyon criteria, to increase the risk of stroke by 85 % during a median 2.1 years of follow-up [25]. However, they focused on elderly patients (≥80 years old) with arterial hypertension without excluding haemorrhagic strokes, which may explain the larger risk [25].

It is to be noted that in our analysis, even the most significant variable reflecting the location of MI (ST elevation in any lead in any of the measured three points) was not proven to associate with IS risk. This corresponds with previous research establishing conflicting results on MI location and IS risk [[17], [18], [19], [20], [21], [22]]. SA in lead V4, on the other hand, seemed to increase IS risk by 13 % (per 1 SD). Even though no certain estimation for reasons behind this can be provided, possible explanations are leftward axis shift (left anterior fascicular block (LAFB)), left (LBBB) or right bundle branch block (RBBB), right ventricular remodelling, LVH (not fulfilling the Sokolow-Lyon criteria) and loss of anterior forces caused by anterior MI.

Increase in lateral TA and TWD were associated with lower risk of IS in our research. These findings on the other way around might be related to strain patterns in LVH or LV dysfunction and, thus, associate with increased risk of IS. Lateral T wave inversion is also associated with increased mortality [27,42]. A recent population-based longitudinal study by Sawano et al. established isolated non-specific ST-segment and T wave abnormalities (NSSTTAs), including lateral T wave inversions, in routine ECGs to increase IS risk by 27 % during a median 9.6 years of follow-up [43]. We found TA in lead V6 to decrease IS risk by approximately 22 % (per 1 SD, median 5.7 years of follow-up) after ACS, which refers to TA lowering being associated with increased IS risk. Gradual lowering of the lateral T wave amplitudes is seen in hypertensive patients when follow-up is performed [44]. This finding may precede the “strain pattern”, which is a sign of LV remodelling in various diseases affecting the LV.

We found long P’WPT in lead V2 to relate to higher IS risk in SR. This could possibly be explained by atriopathy leading to alterations in atrial structure and function, which has been linked to increased IS risk independent of AF [34,45]. Although not identical to P’WPT in lead V2, PTFV1 also reflects the negative component in P wave; PTFV1 is calculated by multiplying the negative P wave amplitude with the duration in lead V1. Whilst not directly comparable, our results align with recent literature demonstrating the association with PTFV1 and IS risk [3,5,7,34,45].

Our research brings forth novel information on the role and magnitude of PVCs or aberrantly conducted complexes in AF/AFL in IS risk, albeit the moderate impact of PVCs independently on IS risk has previously been demonstrated [28]. Our findings of LVH and TA lowering in lead V6 on IS risk are in line with recent literature [[23], [24], [25],^43^]. However, we established SA in lead V4 to increase IS risk, for which we did not find equivalent results in previous studies. Lastly, prolonged P’WPT in lead V2 corresponds to PTFV1, a variable reflecting atrial cardiopathy, and further, IS risk in literature [3,5,7,34,45].

In summary, our results enable novel ways of identifying ACS patients in elevated IS risk simply by analysing the ECG in a clinical setting. These ECG variables could be incorporated into existing risk prediction models to identify patients who may benefit from more aggressive secondary prevention strategies, such as prolonged anticoagulation. Although the results should be externally validated before considering possible clinical applications, the practical possibilities are notable especially considering the utility and wide spread use of the ECG. The role of LVH/LV dysfunction and atriopathy in IS risk was substantial even despite AF/AFL. The results stayed unaltered after adjusting for the CHA_2_DS_2_-VASc Score emphasizing the possibility of complementing the risk score in the future. Hence, the indications for anticoagulative therapy could be broadened outside AF/AFL to possibly include patients with very short atrial high rate episodes or patients suffering cryptogenic strokes who would not otherwise have indication for anticoagulation. Especially PVCs or aberrantly conducted complexes in AF/AFL, LVH and T wave alterations depicting possible strain pattern are easily recognisable ECG variables in the clinical practise and should be integrated into prospective risk prediction models in the future. Atriopathy with multiple possible P wave alterations should be further researched with ambulatory ECG monitoring and combined to the CHA_2_DS_2_-VASc Score. Later on, randomized controlled trials taking into consideration comorbidities and other factors affecting the risk of bleeding are required for verifying the benefit and safety of anticoagulative therapies for patients with increased IS risk following ACS.

One limitation is the retrospective nature of this study affecting the controllability of confounding factors. Since the research time is long (2007–2028 for incident ACS and 2007–2020 for incident IS), there is also possibility for temporal bias, since the medical protocols and the way physicians produce EHR alters over time. For instance, the manner in which IS subtypes are classified has become more precise leading to the validation being more difficult later on. However, there has not been any evident changes over time affecting the relationship of the ECG variables and IS risk.

Another limitation was detecting potential temporary or intermittent alterations since ECG was analysed only momentarily during ACS. Therefore, identifying especially paroxysmal AF/AFL and other alterations in ECGs emerging over time was not possible. Further research on the temporal changes in ECG relating to IS risk is required for better understanding. An evident strength, however, was that the ECG analysis was performed consistently by the automated Marquette™ 12SL™ ECG Analysis Program version 24, although a medical professional may have detected additional findings. This resulted in comprehensive analysis with good repeatability. Automated analysis also enabled uniform analysis to all ECGs and all 725 different variables. To emphasize, the automated ECG analysis was done in single time point after the follow-up time and, thus, there is no difference in the interpretation by the program.

The results are not directly applicable to the general population since the study population consisted of solely ACS patients and external validation is much needed for further interpretation of these results. Even though we included all ACS patients instead of just MI patients, one limitation in this study was the exclusion of patients diagnosed and treated conservatively without coronary angiography [16]. Again, only 8.4 % patients are ruled out of invasive evaluation in our study center due to poor general condition and they have a significant overall mortality reducing the clinical impact of possible secondary preventive measures altogether [46]. In addition, possible unmeasured confounders including socioeconomic status and lifestyle factors were not accounted for in the analyses. Moreover, genetic variability was limited, as 93 % of the Finnish population has a Caucasian background ^47^.

We controlled our analysis for potential false positive results with the Bonferroni method, which is quite a conservative statistical method and, thus, ensured repeatability of our results even if sensitivity was attenuated. Another advantage is that the KARDIO registry is actively maintained and reliable for accurate patient selection. In addition, all incident IS events were identified by an in-depth review of EHRs (including written death certificates) by medical professionals. Due to the centralized nature of Finnish health care (i.e. private hospitals do not provide acute cardiac or neurological care), the follow-up for possible IS events was also high-quality.

## 5 Conclusions

Our research establishes several different ECG features from a vast range of variables associated with IS risk after ACS, although external validation in an independent dataset still is required. Especially markers demonstrating LV dysfunction/remodelling (lowered and shortened lateral T wave) or LVH and ECG parameters reflecting atriopathy resulting in prolonged anterior P wave deflection associate with IS risk.

## CRediT authorship contribution statement

**Matilda Hurskainen:** Writing – review & editing, Writing – original draft, Visualization, Validation, Software, Methodology, Investigation, Funding acquisition, Formal analysis, Data curation, Conceptualization. **Juho Tynkkynen:** Writing – review & editing, Visualization, Supervision, Software, Methodology, Conceptualization. **Leo-Pekka Lyytikäinen:** Writing – review & editing, Supervision, Software. **Terho Lehtimäki:** Writing – review & editing, Methodology, Funding acquisition. **Kjell Nikus:** Writing – review & editing, Visualization, Methodology. **Jussi Hernesniemi:** Writing – review & editing, Supervision, Software, Project administration, Methodology, Funding acquisition, Formal analysis, Data curation, Conceptualization.

## Funding

This research was supported by 10.13039/501100014438Business Finland research funding (4197/31/2015); the 10.13039/501100002341Academy of Finland funding (322098 and 286284); Competitive State Research Financing of the Expert Responsibility area of Tampere University Hospitals (X51001 and Z60104); Finnish Foundation for Cardiovascular Research; 10.13039/100017726Diabetes Research Foundation of Finnish Diabetes Association; 10.13039/501100000780European Union 10.13039/501100007601Horizon 2020 (755320 and 848146); 10.13039/501100000780European Union research funding (101137278); 10.13039/501100010600Tampere University Hospital Supporting Foundation; Pirkanmaa Cultural Foundation; Tampere University Kalle Kaihari Trust; Aarne Koskelo Trust. The Marquette™ 12SL™ ECG Analysis Program version 24 was provided by Ge Healthcare Ltd. (U.S.).

## Declaration of competing interest

None.
