# Supplementary material for: Electrocardiogram markers predicting ischemic stroke after acute coronary syndrome
Source: Int J Cardiol Cardiovasc Risk Prev. 2025 Aug 22;27:200500. doi: 10.1016/j.ijcrp.2025.200500 (PMC12410464; doi:10.1016/j.ijcrp.2025.200500)
Supplement: Multimedia component 1 [file mmc1.docx]

Supplement table 1. ECG factors in multivariable analysis associating with ischemic stroke after acute coronary syndrome (N=7,760) adjusted for the CHA₂DS₂-VASc Score as a continuous variable without age related variables.

| Variable | HR (95% CI) | p-value | SDH (95% CI) | p-value | % (N) | Mean ± SD |
| --- | --- | --- | --- | --- | --- | --- |
| Sex (male) | 1.10 (0.91-1.33) | 0.325 | 1.15 (0.95-1.40) | 0.150 | 65.8 (5,109) |  |
| Ten beats/minute increase in heart rate | 1.06 (1.00-1.12) | 0.041 | 1.01 (0.96-1.07) | 0.680 |  | 70.23 ± 15.18 |
| CHA₂DS₂-VASc Score | 1.36 (1.28-1.45) | <0.001 | 1.22 (1.15-1.30) | <0.001 |  | 3.31 ± 1.60 |
| Previous or current AF/AFL | 1.38 (1.11-1.71) | 0.003 | 1.32 (1.06-1.64) | 0.013 | 19.4 (1,503) |  |
| AF in the ECG* | 1.32 (0.98-1.78) | 0.065 | 1.32 (0.97-1.79) | 0.077 | 7.5 (585) | |
| LVH by Sokolow‐Lyon criteria | 1.54 (1.14-2.08) | 0.005 | 1.53 (1.13-2.08) | 0.006 | 6.0 (462) | |
| PVCs or aberrantly conducted complexes in AF/AFL | 2.10 (1.31-3.38) | 0.002 | 2.07 (1.26-3.40) | 0.004 | 1.3 (103) | |
| PVCs** | 1.15 (0.79-1.66) | 0.465 | 1.07 (0.74-1.56) | 0.710 | 5.6 (432) | |
| S wave amplitude in lead V4 (mV)† | 1.13 (1.04-1.22) | 0.003 | 1.12 (1.04-1.20) | 0.003 |  | 0.72 ± 0.49 |
| ST level elevation in endpoint in lead aVR  (mm)***† | 1.05 (0.94-1.18) | 0.372 | 0.98 (0.89-1.09) | 0.740 |  | - 0.05 ± 0.54 |
| T wave amplitude in lead V6 (mV)† | 0.80 (0.71-0.90) | <0.001 | 0.77 (0.68-0.88) | <0.001 |  | 0.02 ± 0.20 |
| T wave duration in lead aVL (ms)† | 0.83 (0.76-0.90) | <0.001 | 0.84 (0.78-0.92) | <0.001 |  | 175.70 ± 67.67 |
| Multivariable analysis without present AF/AFL in the ECG (7,100). | | | | | | |
| P wave full area in lead V1 (µV*ms)† | 0.92 (0.84-1.01) | 0.082 | 0.93 (0.84-1.02) | 0.120 |  | - 0.95 ± 8.59 |
| Negative P wave peak time in lead V2 (ms)† | 1.11 (1.01-1.22) | 0.028 | 1.10 (1.00-1.22) | 0.048 |  | 19.89 ± 33.08 |

*Included into the multivariable model without the overlapping variable “Previous or current atrial fibrillation or flutter”.

Previously or during the hospitalization for ACS diagnosed atrial fibrillation or flutter.

**PVCs in ECG without AF/AFL were additionally analysed in the multivariable model replacing the variable “PVCs or aberrantly conducted complexes in AF/AFL”.

***ST elevation was measured in three points: J point, middle point (J point + 1/16 of the average PR interval) and endpoint (J point + 1/8 of the average PR interval).

† HR and SDH values corresponding to one standard deviation increase in exposure variable.

Abbervations: HR, hazard ratio; SDH, subdistribution hazard; SD, standard deviation; ECG, electrocardiogram; AF, atrial fibrillation; AFL, atrial flutter; LVH, left ventricular hypertrophy; PVC, premature ventricular complex; AFL, atrial flutter.
